# Host Volatiles Potentially Drive Two Evolutionarily Related Weevils to Select Different Grains

**DOI:** 10.3390/insects15050300

**Published:** 2024-04-23

**Authors:** Shaohua Lu, Lingfang Zhang, Yujie Lu, Mingshun Chen, Zhengyan Wang

**Affiliations:** 1 School of Food and Strategic Reserves, Henan University of Technology, Zhengzhou 450001, China; shaohualu08@163.com (S.L.); lingfang91@163.com (L.Z.); zywangedu@163.com (Z.W.); 2 School of Grain Science and Technology, Jiangsu University of Science and Technology, Zhenjiang 212100, China; 3 USDA-ARS-PSERU, Kansas State University, Manhattan, KS 66506, USA; mchen@ksu.edu

**Keywords:** *Sitophilus zeamais*, *Sitophilus oryzae*, volatiles, olfactory, grains

## Abstract

**Simple Summary:**

The two closely related maize and rice weevils exhibit differential host preferences among stored maize, wheat, and paddy grains. The maize weevil adults prefer to select maize, followed by paddy and wheat, while rice weevil adults mainly migrate towards wheat. The 2-ethylhexanol, piperitone, and (+)-Δ-cadiene are the major components in volatiles from both maize and wheat, but the abundance of these chemicals is much lower in maize than in wheat. The volatile limonene was only detected in paddy. The 2-ethylhexanol, piperitone, and (+)-Δ-cadiene were all attractive to both weevils, whereas limonene was attractive only to rice weevils. The different volatile profiles among the grains and the sensitivity of the two pest species towards these volatiles may explain the behavioral differences between maize and rice weevils in selecting host grains. The variance in sensitivity of maize and rice weevils towards host volatile chemicals with abundance differences is likely a determinant driving the two insect species to migrate towards different host grains.

**Abstract:**

The *Sitophilus zeamais* (maize weevil) and *Sitophilus oryzae* (rice weevil) are two insect pests that have caused huge economic losses to stored grains worldwide. It is urgent to develop an environmentally friendly strategy for the control of these destructive pests. Here, the olfactory-mediated selection preference of the two weevil species to three stored grains was analyzed, which should help establish a pull–push system in managing them. Bioassays showed that maize weevil adults prefer to select maize, followed by paddy and wheat, while rice weevil adults mainly migrate towards wheat. Volatile analyses revealed that 2-ethylhexanol, piperitone, and (+)-Δ-cadiene are the major components in volatiles from both maize and wheat, but the abundance of these chemicals is much lower in maize than that in wheat. The volatile limonene was only detected in paddy. Y-tube bioassays suggest that 2-ethylhexanol, piperitone, and (+)-Δ-cadiene were all attractive to both weevils, whereas limonene was attractive only to rice weevils. Overall, maize weevil appeared more sensitive to the tested volatiles based on having much lower effective concentrations of these volatiles needed to attract them. The differences in volatile profiles among the grains and the sensitivity of the two species towards these volatiles may explain the behavioral differences between maize and rice weevils in selecting host grains. The differences in sensitivity of maize and rice weevils towards host volatile components with abundance differences are likely determinants driving the two insect species to migrate towards different host grains.

## 1. Introduction

*Sitophilus zeamais* Motsch. (maize weevil, Coleoptera: Curculionidae) and *Sitophilus oryzae* L. (rice weevil, Coleoptera: Curculionidae) are two storage product pest species with similar morphology. Initially, they were thought to be two different races of the same species [1,2,3]. However, archaeological records combined with mitochondrial and nuclear ribosomal gene sequence analyses suggest that they evolved into two different species about 8.7 million years ago [2]. Genetic studies revealed that the two weevils differ only in 2 out of 22 chromosomes [4]. Genetic similarity between the two weevils determines their morphological and behavioral similarity. However, maize and rice weevils do exhibit distinct preferences in their choice of host grains. 

Maize and rice weevils have spread through grain trading for centuries and have become a global threat to stored products [3,5,6]. To reduce grain damage caused by these two weevils, fumigation has been widely used for management [7,8,9]. However, the continuous application of chemical fumigants has resulted in the development of insecticide resistance [10,11,12], resulting in reduced effectiveness over time. Recent data suggest that both maize and rice weevils have developed high resistance to commonly used insecticides, including phosphine and pyrethroids [4,5,10,11]. Fumigation along with insecticide application also causes public concerns for food safety and insecticide residues. There is a need for developing environmentally friendly alternative pest control strategies.

Attractants and repellents could be better choices to address the side effects of insecticides [13,14]. Designing attractants and repellents for pests is based on the understanding of their olfactory mechanisms for locating food sources or mates by scent [15,16]. The active ingredients of these attractants and repellents include sex pheromones, alarm pheromones, and specific flavors released by food sources, among others [17,18]. For example, Guarino et al. [19] reported that high emissions of α-ionone and β-ionone in volatiles of *Capsicum annuum*, *C. frutescens*, and *C. chinense* help attract the cigarette beetle, *Lasioderma serricorne* F. (Coleoptera: Anobiidae). Basile et al. [20] indicated that essential oils of *Calendula incana* subsp. maritima and *Laserpitium siler* subsp. siculum show remarkable repellency to the following four stored product pests: *Sitophilus oryzae* L., *Lasioderma serricorne* (F.), *Necrobia rufipes* (De Geer, 1775), and *Rhyzoperta dominica* (Fab.). By studying the olfactory-mediated migration behavior of pest insects, we can get insights into the molecular basis for the development of attractants or repellents. Previous studies have documented that maize weevils prefer to feed polished rice and wheat, followed by maize and paddy [21]. However, the feeding preference of maize weevil can vary with the varieties of grains [21,22].

Based on our laboratory observations, although maize and rice weevils share morphological and host similarities, the two insects select different grains to feed when different grains are available. Namely, maize weevil prefers maize compared with wheat and paddy, while rice weevil prefers wheat among the three grains. Odors from hosts and the environment affect the foraging behavior of insects profoundly. In this study, we first analyzed and compared the volatile profiles of maize, wheat, and paddy grains. Then, we selected the major components of the volatiles to analyze their impact on the host selection of these two weevils. Here we report our initial results on the impact of specific volatiles, selected based on the similarities and differences in volatile profiles among the three grains, on the host selection behavior of the maize and rice weevils. The identified compounds showing attractive effects can be utilized in the design of attractants. Here we report our results on the volatile profile differences and their impact on the orientation behavior of maize and rice weevils.

## 2. Materials and Methods

### 2.1. Insects and Cereals

Maize and rice weevil adults were collected from Guang-an Feed Factory (Zhengzhou, China) (34.3° N, 113.0° E) and Zhangjiawan National Grain Reserve Institution (Tongzhou, Beijing, China) (39.5° N, 116.4° E), respectively, and reared in Henan University of Technology greenhouse (Zhengzhou, China) (34.3° N, 113.0° E). Briefly, 25 pairs of maize weevil adults or rice weevil adults were released into a glass bottle (high:10 cm, diameter: 8 cm) separately and reared on 50 mixed grains of wheat (variety: Zhoumai 22), maize (variety: Xianyu 335), and paddy (variety: TP309). The cultures were maintained under a moisture of about 13% relative humidity. The grain mixture was changed every three days. The insect-rearing bottles were placed into an incubator under a temperature of 28 ± 2 °C and relative humidity of 60 ± 5% (light: dark = 16:8). 

### 2.2. Olfactory Bioassays

#### Four-Arm Olfactometer

A four-arm olfactometer was used to assess the preference of maize and rice weevils for different grains as described in Mwando et al. [23]. Five hundred milligrams of wheat, maize, and paddy grains were separately placed in three arms of the olfactometer. The blank arm was used as a negative control. Airflow was kept at 370 mL/min in each arm with a vacuum pump. Female adults were selected based on examining genitalia and individually released into the center of the olfactometer. A total of 120 female adults were included in each replicate and five replicates were carried out. The positions of different grains were exchanged per 50 insects. Insects crossing into the distal half of the selection arm within 10 min were regarded as a choice. Otherwise, it was recorded as no choice.

### 2.3. Volatile Profiles Analyses

#### 2.3.1. Isolation and Concentration of Volatiles

Volatile profiles of maize, wheat, and paddy were analyzed with the headspace solid-phase microextraction (HS-SPME) method coupled with gas chromatography–mass spectrometry (GC-MS). Specifically, 10 g of wheat, maize, and paddy were placed into 20 mL headspace vials separately (Agilent, Palo Alto, CA, USA). The vials were sealed using crimp-top caps with tetrafluoroethylene (TFE)-silicone headspace septa (Agilent). At the time of solid-phase microextraction (SPME) analysis, each vial was placed at 80 °C for 5 min, then a 120 μm divinylbenzene/carboxen/polydimethylsiloxane fiber (Agilent) was exposed to the headspace of the sample for 30 min [24,25]. Six replications were carried out for each cereal.

#### 2.3.2. Gas Chromatography–Mass Spectrometry Conditions

Analyses of gas chromatography (GC) coupled with mass spectrometry (GC-MS) (Model: QP2010 ultra; Shimadzu, Kyoto, Japan) were carried at 250 °C injection port temperature for 2 min in a splitless mode. The identification and quantification of volatile organic compounds (VOCs) were carried out on the GC-MS equipped with a 30 m × 0.25 mm × 0.25 μm DB-5MS (5% phenyl-polymethylsiloxane) capillary column. Helium was used as the carrier gas at a linear velocity of 1.2 mL/min. The injector temperature was kept at 250 °C and the detector temperature was kept at 280 °C. The oven temperature was set as follows: 8 °C increase per minute from 50 °C to 125 °C, constant temperature at 125 °C for 3 min, 4 °C increase per minute from 125 °C to 165 °C, constant temperature at 165 °C for 3 min, 10 °C increase per minute from 165 °C to 250 °C, and constant temperature at 250 °C for 5 min. Mass spectra were recorded in electron impact ionization mode at 70 eV. Mass spectra were scanned in the range m/z 50–500 amu at 1 s intervals. Identification of volatile compounds was conducted by comparing the mass spectra with the data system library (National Institute of Standards and Technology, NIST 14.0) [26,27]. A series of n-alkanes (C6-C30) was used for the determination of the linear retention index based on the method in van Den Dool and Dec. Kratz [28]. 

### 2.4. Y-Tube Olfactory Bioassay

Based on the volatile analyses, four chemicals with relatively high concentrations in the volatile profiles were selected for Y-tube olfactory bioassay. The bioassay methods were as described by Zhang et al. [26]. Briefly, the Y-tube olfactometer consisted of a glass tube with a 2.5 cm internal diameter and Y-section arms with lengths of 15 cm. There was a 60° angle between the two branches of the Y. The single arm of the “Y” was 20 cm from the junction. The compounds (+)-Δ-cadiene, piperitone, 2-ethylhexanol, and limonene (purity > 99%) were obtained commercially (Toronto Research Chemicals, Toronto, ON, Canada). The chemicals were diluted into a series of concentrations of 0.035, 0.35, 3.5, 35 and 350 mmol/L with paraffin oil. Paraffin oil alone was used as a control. One hundred microliters of diluted chemicals were sprayed onto a 2 × 3 cm filter paper. Two bottles with a piece of cartridge paper in each were connected to the two ends of the Y-tube. Air flow into the arms was 0.3 L/min. When an insect crossed the halfway point of an arm within 3 min, it was regarded as a choice. Forty adults were tested individually and set as one replicate. Three replicates were conducted in each observation. The number of insects in each group was transformed into percentages for difference analyses. Attraction index (Ai) was calculated to evaluate the attractiveness of a chemical with Formula (1) as follows:

The Ai was calculated by Formula (1):

Ai = (T − C)/N(1)

T: the number of insects attracted to a chemical; C: the number of insects that migrated towards the chemical in the control; N: the total number of tested insects.

### 2.5. Data Analyses

One-way ANOVA was carried out to calculate the differences in the preference of insects for four cereals and the GC signal intensity values. Independent *t*-tests were conducted to analyze selection differences between chemical and control insect groups (*p* < 0.05). 

## 3. Results

### 3.1. Olfactometer Bioassays

**Figure 1:**
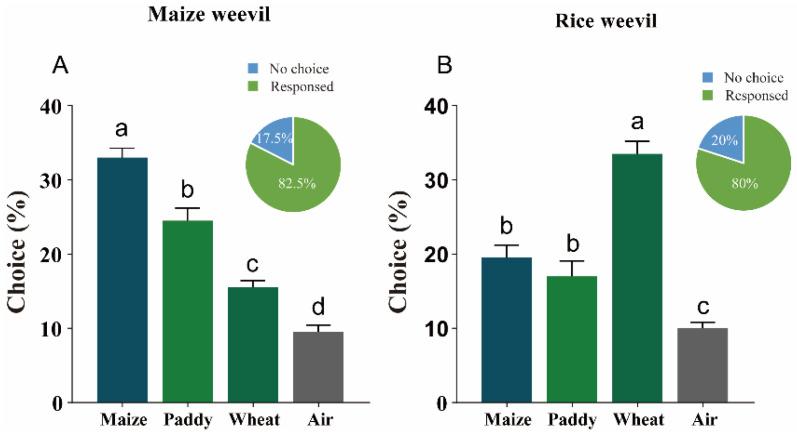
**Preference of maize and rice weevils to different cereals.** Olfactory bioassays of maize weevil (**A**) and rice weevil (**B**) to maize, paddy and wheat compared with clean air. The numbers of insects selecting different grains were transformed into percentages and then were used to calculate differences (one-way ANOVA, *p* < 0.05). Columns showed the percentages of weevils preferring different grains, while pie charts showed the percentages of total weevils making a choice and no preference.

During olfactory bioassays, 82.5% maize weevils and 80% rice weevils made a choice when confronted with different grains. Specifically, over 33% of maize weevil adults selected maize, ~25% selected paddy, ~16% selected wheat, and ~9% migrated to the air control (Figure 1A). On the other hand, 80% of rice weevils chose this test, including 34% of rice weevil adults migrated to wheat, 19% to maize, 17% to paddy, and 10% to air control (Figure 1B). Only 10% of tested insects did not show any host preference in the corresponding assays.

### 3.2. Volatile Profiles among Different Grains

**Figure 2:**
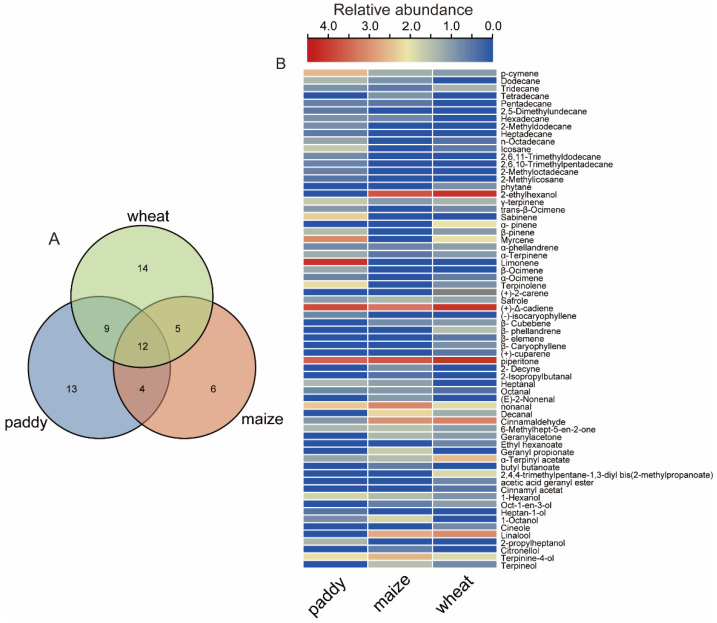
**Characterization of volatile compounds in three grains.** (**A**) Venn plot of volatile profiles. (**B**) Heatmap of identified chemicals in three groups; the colors show the relative abundance of volatiles.

A total of 66 chemicals were detected in volatile analyses of paddy, maize, and wheat, including 16 alkanes, 20 terpenes, 11 alcohols, 7 aldehydes, 7 esters, and 5 other chemicals. Among the three grains, 38 volatiles were detected in paddy, 40 in wheat, and 27 in maize (Figure 2A; Table S1). Twelve (about 18.2% of 66) volatiles were detected in all three grains. The 12 commonly detected chemicals include 4 terpenes (γ-terpinene, α-phellandrene, α-terpinene, and (+)-Δ-cadiene), 2 alcohols (1-hexanol, terpinine-4-ol), 2 alkanes (p-cymene, tridecane), and 2 ketones (piperitone, 6-Methylhept-5-en-2-one) (Figure 2A; Table S1). There were 21, 16, and 17 volatiles detected in paddy and wheat, paddy and maize, and maize and wheat, respectively (Figure 2A). There were 14, 13, and 6 volatiles detected only in wheat, paddy, and maize (Figure 2A). 

**Figure 3:**
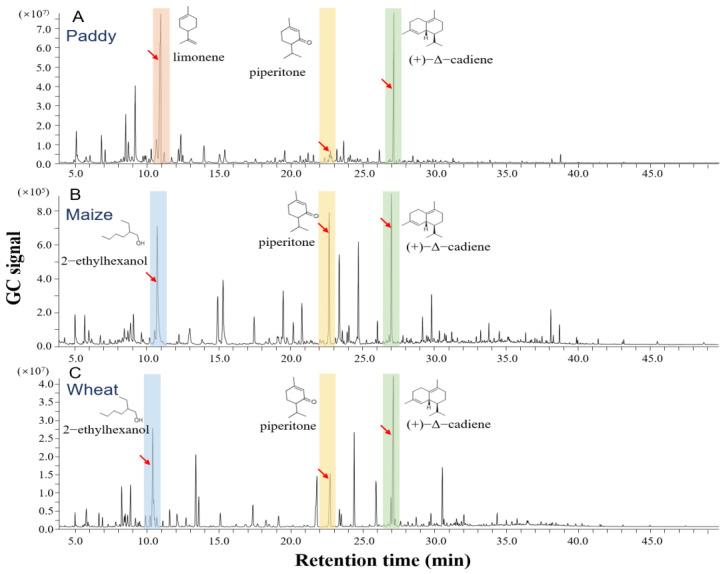
**Gas chromatography profiles of headspace volatiles from different grains.** GC signal intensity represents the relative abundance of volatiles. (**A**) GC signals from paddy. (**B**) GC signals from maize. (**C**) GC signals from wheat. Different colors represent different volatiles. The red arrows represent the peaks of principal chemicals.

**Figure 4:**
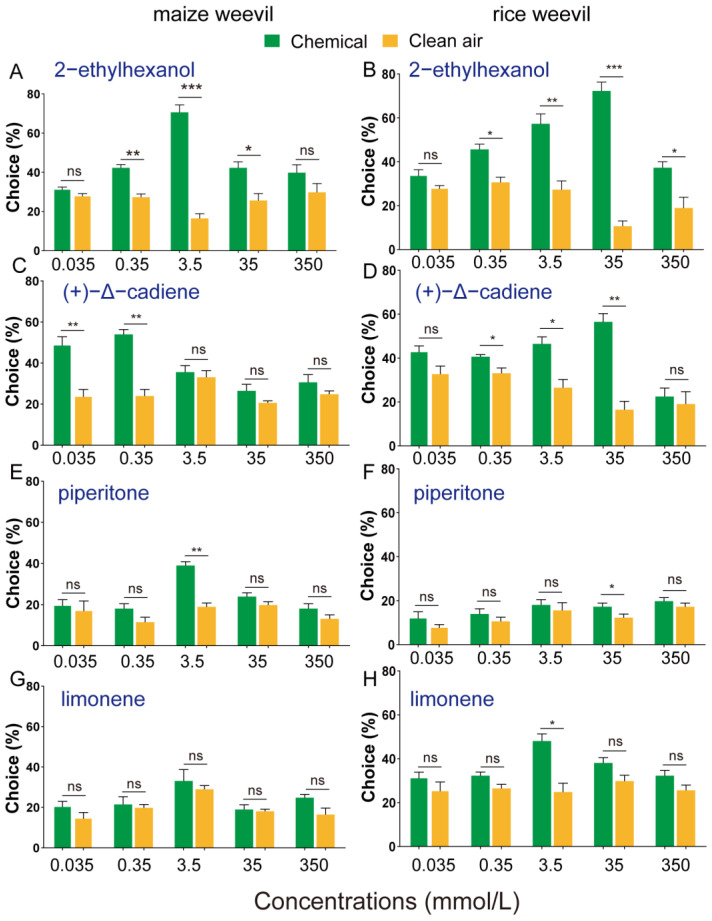
**Behavioral response of maize weevil to different chemicals**. The left part (subfigure **A**,**C**,**E**,**G**) presents the olfactory responses of maize weevil per compound, while the right part (subfigure **B**,**D**,**F**,**H**) presents the selection preference of rice weevil adults to various compounds. The percentage of insects in each group was used to calculate the difference with an independent *t*-test (*p* < 0.05). Forty insects were included in each replicate and three replicates were carried out. “ns” means *p* > 0.05, “*” means *p* < 0.05, “**” means *p* < 0.01, and “***” means *p* < 0.001.

The similarities and differences in the abundance of detected volatiles are shown in Figure 2B. The two chemicals that were most abundant among all three grains are (+)-Δ-cadiene and piperitone. The compounds 2-ethylhexanol, linalool, and cinnamaldehyde are also among the most highly abundant chemicals in maize and wheat but not in paddy. On the other hand, limonene, myrcene, and p-cymene are among the most abundant volatiles in paddy but with very low abundance in wheat and maize. The content of 2-ethylhexanol, piperitone, and (+)-Δ-cadiene was higher in maize than those of other detected chemicals. The two principal volatile chemicals in wheat are (+)-Δ-cadiene and 2-ethylhexanol (Figure 3A and Figure 4). Compared with paddy, maize and wheat showed more similar volatile components. However, the concentrations of several main chemicals are higher in wheat than in maize (Table 1), including 2-ethylhexanol, piperitone, and (+)-Δ-cadiene. Based on olfactometer bioassays and chemical analyses, it appeared that the maize and rice weevils may respond differently to those chemicals that are either uniquely present in different grains or with different abundances. To test these possibilities, Y-tube bioassays were carried out.

### 3.3. Olfactory Attraction of Selected Volatiles to Maize and Rice Weevils

**Figure 5:**
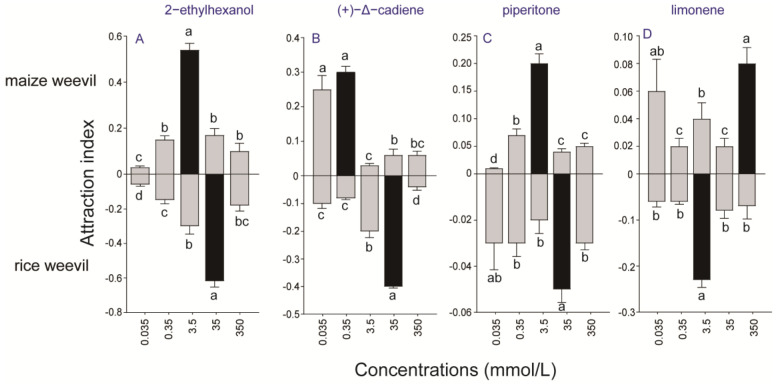
**Attraction index of maize and rice weevils to different compounds.** The absolute value of each column represents the attraction index in each group. (**A**) Attraction indices of maize and rice weevils to different concentrations of 2−ethylhexanol. (**B**) Attraction indices of maize and rice weevils to different concentrations of (+)−A−cadiene. (**C**) Attraction indices of maize and rice weevils to different concentrations of piperitone. (**D**) Attraction indices of maize and rice weevils to different concentrations of limonene. The difference among different groups was calculated by one-way ANOVA (*p* < 0.05). Different letters in the upper part or lower part indicate that there is a significant difference in the comparison.

To evaluate the effects of individual chemicals on the olfactory behavior of maize and rice weevils, Y-tube olfactometers were used to observe the behavioral responses of insects to individual chemicals. As shown in Figure 4A–D, both maize and rice weevil adults showed a choice towards 2-ethylhexanol and (+)-Δ-cadiene, but maize weevil adults responded to the chemicals at much lower doses. On the other hand, piperitone and limonene were less effective or had no impact at all on attracting either maize or rice weevils (Figure 4E–H). Attraction indexes were calculated to evaluate the attractiveness of these four chemicals to the two weevils. The most attractive concentration of (+)-Δ-cadiene to maize weevils is 0.35 mmol/L, which is 100 times lower than that to rice weevils (35 mmol/L) (Figure 5B). Similar phenomena were also found with piperitone (Figure 5C). Limonene is attractive to rice weevils at 3.5 mmol/L but not to maize weevils at the same concentration (Figure 5D).

## 4. Discussion

Maize and rice weevils are destructive pests of stored grains. Even though these two species are closely related and indistinguishable morphologically, each species has its preferred hosts [4]. Both maize and rice weevils can survive on any grain if no choice is available, but each species does better in terms of growth and development on its preferred host [29]. Maize weevil prefers maize while rice weevil prefers wheat if choices are available. It has been long known that insects use chemical cues to locate food sources and mates [16,30,31,32,33]. Volatiles from hosts and the chemosensory system in insects are crucial in plant–insect interactions [34,35,36]. Volatiles from the products made from maize, wheat, rice and other grains have been studied relatively extensively [37,38,39]. However, these studies are in the context of food smell and taste. Volatiles from these grains under storage conditions that attract maize and rice weevils have not been documented. In this study, we systematically analyzed and compared the volatile profiles of stored maize, wheat, and paddy grains. We found similarities and differences in volatile composition and intensity among these three grains. The differences in volatile composition and intensity among different grains may be responsible for the difference in their attraction to maize and rice weevils. Our data have been deposited in public databases and shall be useful for future studies in this field. 

One of the differences in the volatile profiles between maize and wheat is that the abundance of (+)-Δ-cadiene, 2-ethylhexanol, and piperitone in maize was significantly lower than that in wheat. Interestingly, rice weevil is only sensitive to high concentrations of these three volatiles, whereas maize weevil can sense much lower concentrations of the compounds. Therefore, the differences in the abundance of (+)-Δ-cadiene, 2-ethylhexanol, and piperitone between maize and wheat may explain the attraction of corn to maize weevil and the preference of rice weevil to wheat. Further studies are needed to clarify the relationship between the observed differences in volatiles and host attraction to maize and rice weevils. Another difference among the volatiles from maize, wheat and paddy is that the abundance of the volatile limonene was very high in paddy. It has been reported that limonene is lethal to maize weevil at the dose of 9.93 μL/L [40]. However, limonene, at lower concentrations, is attractive to maize weevil. This implied that limonene dose-dependently affects the behavior of maize weevil. In our study, about 80% of tested maize weevils did not show migration towards paddy. Limonene may have some kind of repulsion effect on maize weevils. 

The perception of odors from hosts is mediated via the chemosensory system of insects, which includes OBPs, chemosensory proteins, odorant receptors, ionotropic receptors, and sensory neuron membrane proteins [30,41]. The differences in host selection and odor sensitivity between maize and rice weevils are likely determined by the differences in the chemosensory systems between these two insect species. Unfortunately, very little is known about the chemosensory systems of these two insects. A recent transcriptomic analysis has identified several genes encoding putative OBPs [42]. Similar research needs to be carried out on the identification of other odor perception-related genes. Identification of odor perception genes is also needed in rice weevils. Only then the similarities and differences between the odor-perception systems of maize and rice weevils can be compared and their roles in differential host selection can be tested. The olfactory sensory difference has been well studied in other related species, such as *Polistes fuscatus* and *P. metricus*, the generalist *Helicoverpa armigera* (Hübner), and the specialist *Helicoverpa assulta* (Guenée) [33,43]. Orsucci et al. [44] reported that the two related pest species, European corn borer (*Ostrinia nubilalis* Hbn.) and adzuki bean borer *Ostrinia scapulalis* (Walker) (Lepidoptera: Crambidae), adapted to different host plants and environments with changes in chemosensory repertoire. This means that the physical traits of organisms are dynamically regulated and thus organisms can adapt to different conditions. Therefore, olfactory response variance between maize and rice weevils is also possibly associated with agricultural settings and food resource changes.

In summary, the two closely related maize and rice weevils exhibit differential host preferences among stored maize, wheat, and paddy grains. Systematic analyses and comparisons have been carried out on the volatiles of these grains. Potential candidate volatiles responsible for different host preferences have been selected and initially tested. The differential response of maize and rice weevils to the volatiles 2-ethylhexanol, (+)-Δ-cadiene, and limonene makes these odorants potential determinants for host selection between these two insects. Further analyses of more volatiles considered individually and in combination are needed to identify chemicals that have practical uses in making traps or repellents for these weevil pests.

## 5. Conclusions

The different volatile profiles among the grains and the sensitivity of the two pest species towards these volatiles may explain the behavioral differences between maize and rice weevils in selecting host grains. The variance in sensitivity of maize and rice weevils towards host volatile chemicals with abundance differences are likely determinants driving the two insect species to migrate towards different host grains.

## Figures and Tables

**Table 1:** Relative abundance of four main volatiles among the three grains ^1^.

Chemicals	Paddy		Maize		Wheat	
	Intensity	RI	Intensity	RI	Intensity	RI
2-ethylhexanol	-	-	5.88 ± 0.23 ***	1055	7.20 ± 0.17	1062
limonene	16.71 ± 3.84	1068	-	-	-	-
(+)-Δ-cadiene	7.86 ± 0.33 a	1841	5.91 ± 0.17 b	1824	7.62 ± 0.21 a	1923
piperitone	2.94 ± 0.15 c	1565	5.24 ± 0.29 b	1467	7.17 ± 0.26 a	1571

^1^ Notes: The peak area integral was transformed with log10 for the statistical analysis of differences. Data = mean ± standard error. “-” means that this chemical was not identified in this group. “***” means *p* < 0.001. RI = retention index. Different letters indicate a significant difference between various comparisons. One-way ANOVA and independent *t*-test were used to calculate the difference (*p* < 0.05).

## Data Availability

All data generated or analyzed during this study are included in this published article.

## Supplementary Materials

The following supporting information can be downloaded at https://www.mdpi.com/article/10.3390/insects15050300/s1, Table S1: Volatile profiles of different grains.
