# Supplementary material for: Host Volatiles Potentially Drive Two Evolutionarily Related Weevils to Select Different Grains
Source: Insects. 2024 Apr 23;15(5):300. doi: 10.3390/insects15050300 (PMC12365906; doi:10.3390/insects15050300)
Supplement: Supplementary file 1 [file insects-15-00300-s001.zip › insects-2972203-SI.pdf]

insects MDPI

Table S1. Volatile profiles of different grains.

Chemicals	Paddy		Maize		Wheat	
	Relative Content (%)	Retention Index	Relative Content (%)	Retention Index	Relative Content (%)	Retention Index
p-cymene	4.97 ± 1.55	1057	1.38 ± 0.52	1043	1.01 ± 0.26	1055
Dodecane	1.60 ± 0.66	1112	0.83 ± 0.36	1602	0	-
Tridecane	0.83 ± 0.34	1123	0.46 ± 0.41	1835	1.49 ± 0.56	1127
Tetradecane	0	-	0.85 ± 0.37	1994	0	-
Pentadecane	0.55 ± 0.21	1378	0.43 ± 0.21	2016	0	-
2,5-Dimethylundecane	0.46 ± 0.17	1400	0	-	0	-
Hexadecane	0.80 ± 0.26	1458	0.66 ± 0.26	2125	0	-
2-Methyldodecane	0.75 ± 0.26	1502	0	-	0	-
Heptadecane	0.47 ± 0.21	1544	0	-	0	-
n-Octadecane	1.22 ± 0.26	1590	0	-	0.34 ± 0.14	1995
Icosane	2.16 ± 0.32	1616	0	-	0.48 ± 0.16	2261
2,6,11-Trimethyldodecane	0.72 ± 0.29	1635	0	-	0	-
2,6,10-Trimethylpentadecane	0.58 ± 0.09	1671	0	-	0	-
2-Methyloctadecane	0.57 ± 0.28	1684	0	-	0	-
2-Methylicosane	0.39 ± 0.17	1715	0	-	0	-
phytane	0	-	0	-	0.80 ± 0.29	2510
2-ethylhexanol	0	-	16.50 ± 5.63	1055	12.29 ± 4.59	1062
γ-terpinene	2.16 ± 1.05	921	0.94 ± 0.32	1061	1.52 ± 0.67	1107
trans-β-Ocimene	0.99 ± 0.31	930	0	-	0.70 ± 0.21	931
Sabinene	4.05 ± 1.15	921	0	-	0	-
α-pinene	0	-	0	-	3.26 ± 0.97	981
β-pinene	1.75 ± 0.86	988	0	-	0.97 ± 0.31	988
Myrcene	7.56 ± 0.84	1005	0	-	3.42 ± 1.01	1004
α-phellandrene	0.71 ± 0.34	1028	0.60 ± 0.15	892	1.06 ± 0.17	922
α-Terpinene	1.29 ± 0.25	1044	0.44 ± 0.11	1022	0.94 ± 0.15	1044
Limonene	16.71 ± 3.84	1068	0	-	0	-
β-Ocimene	1.26 ± 0.55	1076	0	-	0	-
α-Ocimene	0.60 ± 0.11	1095	0	-	0.49 ± 0.16	1088
Terpinolene	3.58 ± 1.13	1118	0	-	0.78 ± 0.32	1151
(+)-2-carene	2.35 ± 0.84	1178	0	-	0	-
Safrole	1.04 ± 0.44	1643	1.65 ± 0.72	1634	1.19 ± 0.54	1672
(+)-Δ-cadiene	13.08 ± 5.54	1841	11.49 ± 4.57	1824	16.28 ± 7.05	1923
(-)-isocaryophyllene	0.62 ± 0.21	1936	0	-	0	-
β-Cubebene	0	-	0.73 ± 0.26	2117	0.95 ± 0.34	2304
β-phellandrene	0	-	0	-	1.76 ± 0.58	1065
β-elemene	0	-	0	-	0.41 ± 0.16	1959
β-Caryophyllene	0	-	0	-	0.78 ± 0.24	2039
(+)-cuparene	0	-	0	-	0.40 ± 0.07	1028
piperitone	2.94 ± 1.15	1565	11.94 ± 1.56	1467	8.50 ± 3.56	1571
2-Decyne	0	-	0.80 ± 0.21	1405	0	-
2-Isopropylbutanal	0	-	0.36	832	0	-
Heptanal	1.59	893	1.11	882	0	-
Octanal	0.64	1024	0.92	1003	0.31	1024
(E)-2-Nonenal	0	-	0.98	1115	0	-
nonanal	4.34	1234	7.72	1217	2.8	1186
Decanal	0	-	3.77	1438	1.36	1430
Cinnamaldehyde	0.93	1603	7.19	1564	8.18	1626
6-Methylhept-5-en-2-one	1.57 ± 0.77	996	1.81 ± 0.42	989	1.04 ± 0.27	997
Geranylacetone	0	-	1.60 ± 0.58	1892	1.01 ± 0.23	2117
Ethyl hexanoate	0	-	0	-	0.62 ± 0.19	1017

Geranyl propionate	0	-	2.21 ± 0.13	995	0	-
α-Terpinyl acetate	1.19 ± 0.32	1770	1.71 ± 0.68	1640	4.80 ± 1.21	1840
butyl butanoate	0	-	0.49 ± 0.22	1768	0	
2,4,4-trimethylpentane-1,3-diyl bis(2-methylpropanoate)	0	-	0	-	2.63 ± 0.88	1911
acetic acid geranyl ester	0	-	0	-	0.63 ± 0.23	1932
Cinnamyl acetat	0	-	0	-	0.39 ± 0.21	2107
1-Hexanol	2.46 ± 0.05	859	1.75 ± 0.08	858	0.87 ± 0.23	859
Oct-1-en-3-ol	0	-	0.54 ± 0.19	980	1.02 ± 0.29	990
Heptan-1-ol	0.39 ± 0.11	976	0	-	0	-
1-Octanol	0.75 ± 0.36	1145	2.54 ± 0.38	1115	0	-
Cineole	0	-	0	-	0.71 ± 0.29	1067
Linalool	0	-	5.59 ± 0.24	1144	7.38 ± 3.67	1178
2-propylheptanol	1.56 ± 0.27	1220	0	-	0	-
Citronellol	0	-	0.53 ± 0.22	1388	0	-
Terpinine-4-ol	3.40 ± 0.74	1407	5.05 ± 1.16	1400	2.72 ± 1.08	1346
Terpineol	0	-	1.83 ± 0.67	1417	0.71 ± 0.15	1387

Notes: the relative content was calculated by the peak area of each chemical in GC signal. The percentage of each chemical shows its relative abundance in the volatile profiles. “0” means this compound was not identified in this group.
